# Clinical and Epidemiological Characteristics of Severe Acute Adult Poisonings in French Amazonia: Urgent Need for a Toxicovigilance Monitoring Framework

**DOI:** 10.3390/toxics12030200

**Published:** 2024-03-05

**Authors:** Jean Marc Pujo, Yann Simon, Guy Roger Lontsi Ngoulla, Boubacar Signaté, Rémi Mutricy, Alexis Frémery, Antoine Burin, Bertrand de Toffol, Ibtissem Ben Amara, Stephanie Houcke, Amina Nasri, Dabor Resiere, Hatem Kallel

**Affiliations:** 1 Emergency Department, Cayenne General Hospital, Cayenne 97300, French Guiana; tamac1966@gmail.com (J.M.P.); ynnsimon0363@gmail.com (Y.S.); boubakar.signate@ch-cayenne.fr (B.S.); remi.mutricy@ch-cayenne.fr (R.M.); alexis.fremery@ch-cayenne.fr (A.F.); antoine.burin@ch-cayenne.fr (A.B.); 2 Tropical Biome and Immunopathology CNRS UMR-9017, Inserm U 1019, Université de Guyane, Cayenne 97300, French Guianaamina.nasri@ch-cayenne.fr (A.N.); 3 Intensive Care Unit, Cayenne General Hospital, Cayenne 97300, French Guiana; guy.lontsingoulla@ch-cayenne.fr (G.R.L.N.); ibtissem.benamara@isbs.usf.tn (I.B.A.); stephanie.houcke@ch-cayenne.fr (S.H.); 4 Neurology Department, Cayenne General Hospital, Cayenne 97300, French Guiana; 5 Intensive Care Unit, Martinique University Hospital, Fort de France 97261, Martinique

**Keywords:** epidemiology, acute poisoning, paraquat, toxicovigilance, French Guiana

## Abstract

Background: Acute poisonings (AP) are a significant public health problem, accounting for a high number of emergency department visits and thousands of deaths worldwide. This study aimed to assess the epidemiology of AP in an adult population admitted to Cayenne Hospital (French Guiana) and to investigate the clinical and sociodemographic characteristics. Methods: We conducted a monocentric retrospective study from January 2010 to December 2022, including patients over eighteen years of age who had been admitted to the emergency department of Cayenne Hospital for acute poisoning. Results: We included 425 patients. The median age was 34 years (IQR: 25–47). The sex ratio (M/F) was 0.52. A psychiatric disorder was found in 41.9% of patients. The Poisoning Severity Score (PSS) on admission was 1 or 2 for 84% of patients, and the mortality rate was 3.9%. The main involved toxicants were psychotropic drugs (43.1%), benzodiazepines (34.8%), and paracetamol (25.6%). The most lethal toxic was paraquat (5.2%). Intoxication was due to intentional self-poisoning in 84.2% of cases. Independent factors associated with severe poisoning (PSS 3 or 4) were chloroquine, neuroleptics, or paraquat poisoning; metabolic acidosis; and hyperglycemia (>5.5 mmol/L). The mortality rate was 3.9%, and the most involved toxic in death was paraquat. Conclusion: This study shows the frequent and deadly use of paraquat in APs in French Guiana. Urgent attention should be given to establishing a toxicovigilance monitoring framework and an antipoison center in the region.

## 1. Introduction

Acute poisoning (AP) is defined as adverse health effects resulting from acute (less than 24 h), voluntary, or accidental exposure to a toxic substance, whether from industrial, environmental, chemical, or biological origin or in connection with envenomation [1,2]. Acute poisoning is a frequent cause of emergency department (ED) visits and hospital admissions. It accounts for 1–3% of all ED visits [1] and up to 10% of the caseload [3]. According to the WHO, more than 300,000 people died worldwide in 2013 because of poisoning [4]. In the same year, French anti-poison centers recorded 168,475 cases of toxic exposure, and 43.9% of them were symptomatic [5]. In a more recent report from the WHO published in 2019, the mortality rate attributed to unintentional poisoning (per 100,000 population) was 0.4 in high-income and 2.3 in low-income countries, and it was higher in males than in females [6].

The causes of AP vary across regions depending on the sociodemographic and cultural characteristics of the studied population, the level of development/industrialization of the country, and the presence of a drug prescription policy [2,3,7,8]. Pesticides are the most frequent causes of poisoning in low- and middle-income countries, while household products and specialty drugs are at the top in high-income countries [2,3,7,9,10,11,12]. Indeed, pesticide poisoning is a major global public health challenge predominantly impacting Southern Asia and South and Central America [8,13]. However, in industrialized countries, psychotropics are an increasing cause of poisoning and death [14,15,16,17]. During recent years, the rate of psychotropic agents administered increased by almost 60% [14,16]. This rise was followed by a subsequent increase in poisonings by these drugs. Consequently, prescription of psychotropics may play a role in this poisoning increase [18,19]. Also, gender and age play significant roles in the epidemiology of psychotropic drug poisoning [20,21]. Poisoning is considered severe when requiring close monitoring due to the intrinsic toxicity of the product and the quantity to which the patient has been exposed.

Professionals and health authorities need updated information on local AP characteristics to adapt healthcare professionals’ practices and run specific prevention campaigns [3]. For this, most developed countries have implemented poison and toxicovigilance centers to monitor poisoning cases and advise callers based on regularly updated information. There has yet to be a reliable database of toxic substances responsible for AP in French Guiana.

Our study aimed to assess the incidence of AP among adults managed in Cayenne General Hospital in French Guiana, to depict their etiological spectra, and to describe their clinical and sociodemographic characteristics.

## 2. Materials and Methods

### 2.1. Study Area Description

A cross-sectional retrospective study was carried in the ED of the Cayenne Hospital over 13 years, from January 2010 to December 2022. We included adult patients (aged 18 or over) presenting with acute poisoning, defined according to the 10th version of the International Classification of Diseases (ICD-10) criteria [22]. The diagnosis codes used to select patients were poisoning by drugs and biological substances (T36 to T50) and toxic effects of essentially non-medicinal substances (T51 to T65). We excluded all patients under 18, those presenting with envenomation by animal bite or sting (T63), and those whose records were missing or did not provide sufficient data for analysis. The files were obtained from the medical information department and the Emergency Medical File software and were reviewed by two authors (Y.S. and H.K.). Only cases presenting acute symptomatic intoxication with a “Poisoning Severity Score” PSS ≥ 1 were studied [23].

### 2.2. Data Collection Procedures

We recorded epidemiological and clinical data, including the patient’s sex and age, the personal history (including substance abuse and psychiatric disorders), the date and time of intoxication, the type of toxic substance, the mode of exposure, the clinical and biological parameters on admission to the emergency department, the management protocol, and the length of hospital stay. Metabolic acidosis was defined as alkaline reserve < 23 mmol/L, hypokalemia as a potassium dosage < 3.5 mmol/L, and hyponatremia as sodium dosage < 135 mmol/L. Thrombocytopenia was defined by a platelet count < 150 G/L. The Kidney Disease Improving Global Outcomes (KDIGO) definition was considered for renal impairment [24]. The type of exposure was classified as intentional (drug abuse or voluntary self-poisoning), unintentional (accidental overdose or misuse), or unknown. Toxic agents were classified as pharmaceutical drugs or non-pharmaceutical toxic agents. Toxics were classified according to the product’s indication for use. Combined drug intoxication referred to the involvement of two or more pharmaceutical drugs.

### 2.3. Statistical Analysis

Results are presented as the number of patients for whom data was recorded (Nb), the median and interquartile range (IQR: 1st–3rd quartiles), or numbers with percentages. Initial bivariate statistical comparisons for categorical variables were made using the Chi-square test or Fisher’s exact test. Continuous variables were compared using the Mann–Whitney U test. Multivariate logistic regression was used to identify variables associated with mortality. Correlation between quantitative variables was assessed using linear regression. Non-redundant variables selected by bivariate analysis (*p* ≤ 0.05) and considered clinically relevant were entered into the logistic regression model to assess independent factors associated with severe poisoning (PSS 3 or 4). Statistical analyses were performed using Excel (2010 Microsoft Corporation, Redmond, WA, USA) and IBM SPSS Statistics for Windows, version 24 (IBM Corp., Armonk, NY, USA).

## 3. Results

### 3.1. Sociodemographic Characteristics of the Included Cases

**Figure 1:**
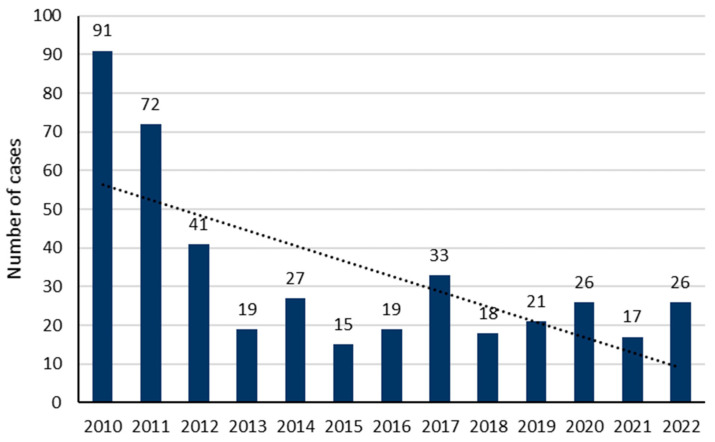
Yearly distribution of poisoning cases included in our study.

**Figure 2:**
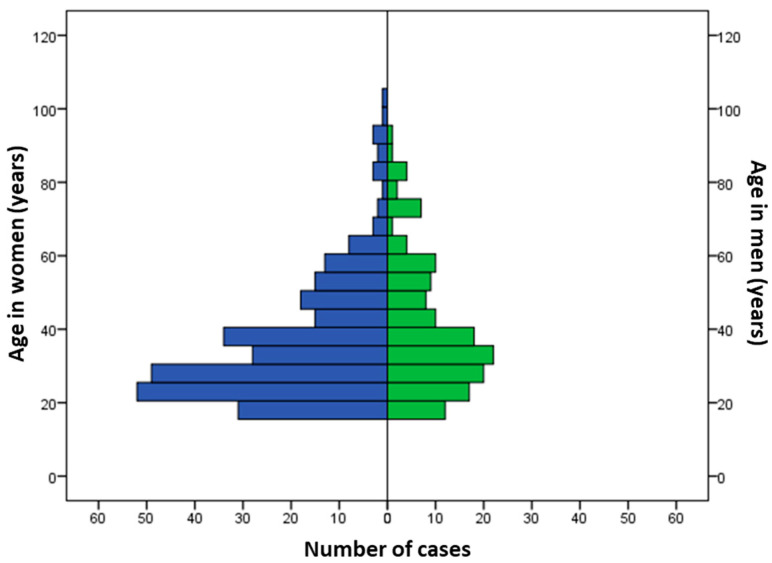
Distribution of cases according to age and gender.

During the study period, 425 patients met our inclusion criteria. The average number of cases was 33 ± 23 per year, with a decreasing trend over the years (*p* = 0.0131; Figure 1). The sex ratio (M/F) was 0.52. The median age was 34 years (IQR: 25–47), but was significantly older in men (36 years; IQR: 27–51) as compared to women (32; IQR: 24–46) (*p* = 0.014; Figure 2). A history of psychiatric disorders was found in 41.9% of cases. Among patients, 357 (84%) were classified as PSS 1 or 2 and 68 (16%) as PSS 3 or 4. Table 1 shows the patients’ epidemiological data and compares the data for the PSS 1 or 2 and PSS 3 or 4 groups.

### 3.2. Implicated Toxic Substances

**Figure 3:**
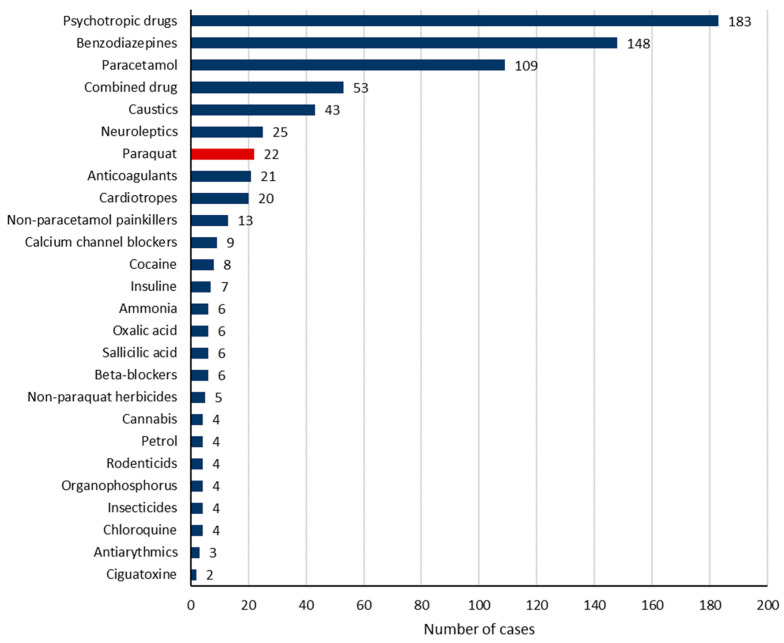
The involved toxic agents.

Intentional self-poisoning was recorded in 84.2% of cases. Pharmaceutical drug intoxication occurred in 78.6% of cases. The most common involved pharmaceutical drugs, alone or in combination, were psychotropics (43.1%), benzodiazepines (34.8%), and paracetamol (25.6%) (Figure 1 and Table 2). Furthermore, the toxic agents were non-pharmaceutical in 24.2% of cases. They encompassed mainly caustic agents in 10.1% of cases, herbicides in 6.4% of cases (including paraquat in 5.2% of cases), cocaine in 1.9% of cases (including crack cocaine in 0.2%), oxalic acid (Rubigine^®^) in 1.4% of cases, ammonia in 1.4% of cases, and rodenticide in 1.4% of cases. Figure 3 and Table 2 show the frequency of the involved toxics.

### 3.3. Clinical Manifestations at Admission to the Emergency Department

The most frequent clinical manifestations were digestive disorders (49.8%), tachycardia (24.6%), bradypnea (30%), and coma (9.7%) (Table 3). Hypothermia was found in 0.9% of patients in the PSS 1 or 2 group and 5.8% of patients in the PSS 3 or 4 group. Tachycardia was found in 23.7% of patients in the PSS 1 or 2 group and 29.9% of patients in the PSS 3 or 4 group. Circulatory shock was found exclusively in 16 PSS 3 or 4 patients. Coma was present in 2.8% of patients in the PSS 1 or 2 group and 45.6% of patients in the PSS 3 or 4 group. The most common biological abnormalities were metabolic acidosis (29.1%), hypokalemia (26.5%), and hyponatremia (12.4%). These abnormalities were more common in the PSS 3 or 4 group than in PSS 1 or 2 (Table 4).

### 3.4. Management of Acute Poisoning Cases

Toxicological screening was performed in 273 patients (64.2%). Activated charcoal was used in 28% of cases. Gastric lavage was used in 11.8% of cases and antidotes in 45%. Mechanical ventilation was used in 22.1% of PSS 3 or 4 patients. Vasopressors were administered in 26.5% of PSS 3 or 4 group patients. Dialysis was used in 4.4% of patients in PSS 3 or 4 (Table 5). The most frequently used antidotes were N-acetyl-cysteine and Flumazenil.

### 3.5. Outcome of Poisoned Cases

Hospital admission was required for 266 patients (62.6%) and ICU admission for 28 (6.6%). The median length of hospital stay was two days (IQR: 2–3). It was 2 days (IQR: 2–3) in patients hospitalized in the ward and 3 days (IQR: 1–6) in those hospitalized in ICU.

Among variables associated with the poisoning severity (PSS 3 or 4), nine were considered relevant and were included in the multivariate analysis: age, acute renal failure, metabolic acidosis, hyperglycemia (>5.5 Mmol/L), intoxication to paracetamol, caustics, chloroquine, neuroleptics, and paraquat. Of them, independent factors associated with severe poisoning were chloroquine (*p* = 0.017; OR: 18.374; 95% CI: 1.665–200.561), neuroleptics (*p* = 0.009; OR: 3.893; 95% CI: 1.409–10.753), and paraquat poisoning (*p* = 0.012; OR: 5.472; 95% CI: 1.452–20.618); metabolic acidosis (*p* = 0.048; OR: 2.109; 95% CI: 1.005–4.424); and hyperglycemia (>5.5 mmol/L) (*p* = 0.049; OR: 2.404; 95% CI: 1.002–5.766). Paracetamol poisoning was inversely associated with severe poisoning (*p* = 0.036; OR: 0.324; 95% CI: 0.113–0.931) (Table 6). Overall, 16 patients died, giving a crude mortality rate of 3.9%. The involved toxics in deceased patients were paraquat in 14 patients, chloroquine in 1 patient, and caustics in 1 patient.

## 4. Discussion

Our study shows that AP in French Guiana involves mainly young female people with a history of psychiatric disorders. Intoxication was related to intentional self-poisoning in most cases. The main involved toxicants were psychotropic drugs, benzodiazepines, and paracetamol. Independent factors associated with mortality were paraquat poisoning, hypokalemia, and acute renal failure. These data provide insights on how to improve the medical management of and mental health care for people with AP in French Guiana. They also emphasize the need for a toxicovigilance monitoring framework to monitor the trends and profiles of acute poisoning cases and the involved toxics.

Acute poisoning is a significant public health problem worldwide. In 2018, the American Association of Poison Control Centers reported 2,099,751 cases of toxic exposure, of which 48.6% involved pharmaceutical drugs [25]. In the United Kingdom, between 2018 and 2019, the National Poisons Information Service reported 170,000 patients hospitalized due to poisoning, representing 1% of all hospital admissions [26]. In Germany, the incidence of AP rose from 1.2% to 1.9% between 2005 and 2012 [27]. In France, a retrospective study using the Poison Centre Information System data in 2013 reported 168,475 exposure cases, with a peak incidence in Corsica of 39.2 cases per 100,000 inhabitants [5]. In Martinique, a retrospective study from 2000 to 2010 reported an incidence of 7.7 severe poisoning cases/100,000 inhabitants [2]. Our study recorded 425 adult poisoning cases over 13 years, with a decreasing incidence trend over the years.

In this study, 65.6% of patients were women, with an average age of 32 years. A history of psychiatric disorders was present in 41.9% of patients. Intentional self-poisoning was recorded in 84.2% of cases, and the most involved toxics were psychotropics, benzodiazepine, and paracetamol. These findings are in line with previous studies showing that intoxicated patients in industrialized countries are generally young women with psychiatric histories [28,29,30,31,32]. However, this result is different from that observed in Martinique, where the male gender was recorded in 57% of AP cases [2].

As far as the implicated toxic agents are concerned, an Australian study showed that tricyclic antidepressants, benzodiazepines, and ethanol were the most involved toxics, and multidrug AP was present in 65% of cases [30]. Conversely, pesticide poisoning, mainly organophosphate poisoning, was the leading toxic cause in developing countries [3,33,34]. An Ethiopian study showed organophosphates (45%) and sodium hypochlorite (22.5%) as the most involved toxics [3]. It is noteworthy that etiologies of AP have experienced notable shifts in recent years, requiring close monitoring of the involved toxics [1,35,36,37]. Also, healthcare professionals managing AP must have advanced skills in clinical toxicology to provide tailored care according to the toxic and the patient’s characteristics [38]. Furthermore, the management strategy must incorporate the pharmacokinetic/pharmacodynamic properties of the involved toxic and consider the availability of specific investigations, antidotes, and rescue techniques [38]. Lastly, healthcare professionals, policymakers, and health authorities must focus on preventive programs based on the most involved toxics and patient characteristics.

Regarding AP outcomes, mortality rates secondary to AP vary considerably among studies and depend essentially on the type of the predominant toxicant and access to care [28,39,40,41]. The rate was 0.9% in South Africa, 1.9% in Sweden, and up to 10% in Martinique [2,28,39]. While lethality from AP in Martinique was the highest in the literature, it may be explained by the inclusion of severely intoxicated patients and the involvement of combined pesticides and pharmaceutical drugs [2]. A multi-source study carried out in Paris in 2010 and 2011, including the Paris Poison Control Centre (CAP); the Lariboisière ED and ICU, which are part of the Organization of Coordinated Emergency Surveillance (OSCOUR) network; the Île-de-France regional pharmacovigilance co-ordination unit; and two toxicology laboratories involved in forensic assessments after lethal intoxication, recorded 9520 cases of intoxication (sex ratio M/F = 0.77), with a mortality rate of 2.18% [42]. Factors associated with mortality were male gender, organ failure, metabolic acidosis, hypokalemia, rhabdomyolysis, and hepatic cytolysis. In our study, the mortality rate was 3.9%, in line with most international references. However, the most lethal toxic in our region was paraquat. Additionally, the toxics associated with severe poisoning were chloroquine, neuroleptics, and paraquat.

According to the WHO, more than 77% of suicides occurred in low- and middle-income countries in 2019. Also, 20% of suicides involved pesticide poisoning and occur mainly in rural areas [43]. In Suriname, half of the suicides and attempted suicides involved pesticides, mainly paraquat [44]. In French Guiana, suicides caused by paraquat generally occurred in remote areas [45]. Paraquat (1,1 dimethyl 4,4′ bipyridylium dichloride) is a harmful non-selective herbicide [45,46]. Despite its high toxicity, it is still widely used worldwide, as are many other plant protection products like glyphosate and organophosphates [46,47]. Given its high toxicity to humans (50–90% mortality following ingestion), Europe banned its use in July 2007 [48,49,50]. However, it remained available in neighboring countries and is illegally imported into French Guiana. In France, in 2008, the “Ecophyto” plan banned 30 active substances contained in pesticides (including paraquat) [51,52]. Despite this prohibition, 23.8% of calls to French Anti-Poison Centers concerning pesticide poisonings between 2012–2016 involved paraquat [51]. The lethal ingested dose of paraquat is 35 mg/kg. Note that a 20 mL sip of Gramoxone^®^ (the commercial paraquat name) equals 57 mg/kg in a 70 kg adult [53]. French Guiana has the highest incidence of Paraquat-induced AP in the EU, with 3.8 cases/100,000 inhabitants/year [45]. Various avenues, therefore, need to be explored, including educating the public and improving product safety (addition of an emetic, a laxative, and an alginate: Gramoxone INTEON) [53,54,55]. In South Korea, paraquat prohibition has led to a 46.1% drop in paraquat AP [56]. In 2020, Brazil banned paraquat because of its acute and chronic toxicity [57]. Accordingly, banning Paraquat in Suriname would be an option due to its harmful effect on the environment, human, and animal health [58]. A One Health approach can help to optimize the net benefits and risks from pesticides and paraquat use on plants, people, animals, and ecosystems [59].

In our study, 41.9% of patients suffered from psychiatric disorders, raising the issue of mental health in the context of AP. Also, there was a close link between mental disorders and intentional self-poisoning (84.2%), as is similar to a Spanish report (83.2%) [60]. One of the leading causes of AP is suicide attempts. In an Indian study, 64% of APs were related to a suicide attempt [33]. In 2018, Santé Publique France reported that intentional drug intoxication was the most frequent mode of suicide (87% of hospital admissions for suicide in women and 75% in men) [61]. In the United States, in 2000, 80% of suicide attempts were associated with AP [62]. The WHO reported 877,000 deaths by suicide in 2003 [63]. In French Guiana, the overall suicide rate is estimated at 7 per 100,000 inhabitants/year, which is lower than in mainland France (16/100,000 inhabitants/year). Still, most cases of suicide are reported in the Amerindian communities of Camopi and Trois-Sauts (113 and 137 deaths/100,000 inhabitants/year). The most frequently used methods are hanging (72%) and AP (18%). Also, voluntary AP accounted for 50% of hospital stays for suicide attempts recorded between 2015 and 2017 in French Guiana [64]. Women account for two-thirds of suicide attempts, with a 3.7 times higher incidence of AP than men. In the Maripasoula municipality, the rate of suicide attempts by paraquat absorption was the highest, at 46% [64]. Albano et al. [8], in a systematic review of toxicological findings of self-poisoning suicidal deaths, found that the most involved substances in low- and middle-income countries with significant agricultural areas were pesticides such as organophosphates and carbamates. In contrast, in high-income countries, the use of illicit drugs and medicines for suicide was more frequent. Collados-Ros et al. [65] reported that most suicides were associated with drug abuse, mainly psychopharmaceuticals. In this study, the authors highlight the vital role of toxic substances in suicidal behaviors. In 2016, an “Equality and Citizenship” bill on the fight against suicide among the young Guyanese population was proposed to the government to create a regional suicide observatory [66]. In 2020, the Regional Agency for Health in French Guiana set up the “Centre Ressource Prévention du Suicide” and the “VigilanS” system. This original and unique platform in France combines monitoring, crisis intervention, recidivism prevention, and training. The “Well-being of the Populations of the Interior of French Guiana,” called the “BEPI program,” complements this policy. In addition to these devices, involving mental health specialists in the emergency care teams could play a crucial role in assessing and managing at-risk and poisoned patients.

The scientific literature reflects the sociodemographic, cultural, geographical, and spatial–temporal variability of APs [64,67,68,69]. All these parameters and the prevalence of APs observed in our study highlight the need for a toxicovigilance center (TVC) dedicated to French Guiana. The aim of toxicovigilance is to monitor the acute or chronic toxic effects to humans of exposure to a natural or synthetic mixture or substance available on the market or found in the environment in order to undertake alert and prevention actions, as stated in the L. 1340-2 article of the French Public Health Code. This activity covers collecting and analyzing information and alerting the public to enable preventive action, according to the R.1341-16 article of the French Public Health Code. Toxicovigilance in France is organized nationally and regionally based on the network of 13 Acute Poisoning and Toxicovigilance Centers (APTVCs) spread throughout the country. The APTVCs respond 24/7 to any request for risk assessment or advice on the diagnosis, prognosis, and treatment of human intoxication. Each call is entered into a database. The latter can be used for toxicovigilance by assessing the involved toxins and proposing appropriate action. Accordingly, TVCs organize thematic networks at the local level and joint surveillance with the Health Monitoring Institute (InVS) at the national level. It contributes as an investigation reporter or data producer. Thereafter, data are analyzed by the “Cellule interrégionale d’épidémiologie” (CIRE).

Overall, there is an urgent need for a dedicated toxicovigilance monitoring framework in the French overseas departments [2]. The French West Indies and French Guiana depend on the APTVC in Paris, while Réunion Island and Mayotte depend on the APTVC in Marseille. In addition, there have been attempts to develop local and regional structures, with the Indian Ocean Toxicovigilance System (IO-TVS) in 2006, which allowed the epidemiological situation of pesticide-induced AP in Reunion Island to be described. In 2009, a joint mission by the InVS, the “CIRE Antilles-Guyane,” the Anti-Poison Center in Paris, and the TVC in Grenoble recommended setting up a TVC in the French West Indies departments. Its implementation in 2014 enabled the identification of specific intoxications in Guadeloupe. These examples demonstrate the value of implementing a TVC in French Guiana for the purpose of improving knowledge of APs and developing activities targeting toxicological themes. It is a priority in French Guiana to investigate traditional medicine’s beneficial or harmful effects, as well as intoxications by herbicides (Paraquat) and those attributable to local fauna. Moreover, no regional or inter-regional register exists to identify APs or to enable descriptive analyses. Furthermore, AP poses a significant public health challenge in the French Territories in the Americas (FTA), which experience a notable prevalence of rural and domestic poisonings. Consequently, it is imperative to establish dedicated Poison and Toxicovigilance Centers within these departments to enhance the quality of care and facilitate the prompt identification of individuals affected by toxic substances. Moreover, a deeper understanding of the impact of pesticide use in French Guiana and the FTA on human, animal and environment is needed within the One Health concept.

Our study has several limitations due to its retrospective, monocentric design, and focuses mainly on symptomatic patients. However, Cayenne Hospital is the referral healthcare facility in French Guiana, managing more than 50% of ED visits and most severe poisoning cases. Furthermore, this is the first large-scale epidemiological study shedding light on the peculiarities of AP in the adult population in French Guiana. It confirms the burden of AP as a neglected public health problem and the urgent need to set up a dedicated toxicovigilance monitoring framework in French Amazonia and an antipoison center in the French Territories in the Americas.

## 5. Conclusions

This study enabled us to describe the epidemiology of adult poisoning cases attending the ED of Cayenne Hospital over 13 years. Our results demonstrate the seriousness of paraquat-induced AP. This up-to-date toxicological knowledge is essential to guide emergency physicians in diagnosing and managing poisoned patients. The fight against suicide and the monitoring of mental disorders must form an integral part of the public health policy in French Guiana. Also, there is an urgent need to equip French Guiana with a toxicovigilance system and the FTA with a dedicated APC to establish a local register of acute and chronic intoxications. Prospective multicenter studies in South America, the Amazon region, and the FTA are needed in order to better understand the epidemiology of acute poisonings.

## Figures and Tables

**Table 1:** Epidemiological characteristics of poisoning cases.

	Total		PSS 1 or 2		PSS 3 or 4		
	Nb	Result	Nb	Result	Nb	Result	*p*
Male sex	425	146 (34.4%)	357	117 (32.8%)	68	29 (42.6%)	0.116
Age (years)	425	34 (25–47)	357	32 (24–45)	68	41 (31–58)	0.001
Personal medical history							
Psychiatric disorders	425	178 (41.9%)	357	152 (42.6%)	68	26 (38.2%)	0.506
Voluntary medical intoxication	425	145 (34.1%)	357	123 (34.5%)	68	22 (32.4%)	0.738
Alcohol abuse	425	54 (12.7%)	357	46 (12.9%)	68	8 (11.8%)	0.799
Poisoning characteristics							
Pharmaceutical drug	425	334 (78.6%)	357	286 (80.1%)	68	48 (70.6%)	0.079
Combined drug	425	55 (12.9%)	357	43 (12%)	68	12 (17.6%)	0.207
Caustic	425	70 (16.5%)	357	50 (14%)	68	20 (29.4%)	0.002
Alcohol	425	53 (12.5%)	357	45 (12.6%)	68	8 (11.8%)	0.848
Intentional self-poisoning	425	358 (84.2%)	357	305 (85.4%)	68	53 (77.9%)	0.120
Unintentional poisoning	395	321 (81.3%)	331	274 (82.8%)	64	47 (73.4%)	0.080
Management							
Hospitalization	425	265 (62.4%)	357	215 (60.2%)	68	50 (73.5%)	0.038
Hospital length of stay (days)	425	2 (2–3)	357	2 (2–3)	68	3 (2–5)	0.012

Values indicate the number of patients for whom data were recorded (Nb), the median and interquartile range, or numbers and percentages.

**Table 2:** Pharmaceutical and non-pharmaceutical toxic agents recorded in poisoning cases.

	Total		PSS 1 or 2		PSS 3 or 4		*p*
Toxic Agent	Nb	Result	Nb	Result	Nb	Result	
Pharmaceutical (drug)	425	322 (75.8%)	357	277 (77.6%)	68	45 (66.2%)	0.044
Psychotropic drugs	425	183 (43.1%)	357	152 (42.6%)	68	31 (45.6%)	0.646
Benzodiazepines	425	148 (34.8%)	357	122 (34.2%)	68	26 (38.2%)	0.519
Analgesics	425	122 (28.7%)	357	113 (31.7%)	68	9 (13.2%)	0.002
Paracetamol	425	109 (25.6%)	357	102 (28.6%)	68	7 (10.3%)	0.002
Neuroleptics	425	25 (5.9%)	357	17 (4.8%)	68	8 (11.8%)	0.024
Anticoagulants	425	21 (4.9%)	357	19 (5.3%)	68	2 (2.9%)	0.551
Cardiotropic drugs	425	20 (4.7%)	357	14 (3.9%)	68	6 (8.8%)	0.080
Calcium channel blockers	425	9 (2.1%)	357	7 (2%)	68	2 (2.9%)	0.641
Insulin	425	7 (1.6%)	357	4 (1.1%)	68	3 (4.4%)	0.085
Beta-blockers	425	6 (1.4%)	357	5 (1.4%)	68	1 (1.5%)	1.000
Salicylic acid	425	6 (1.4%)	357	5 (1.4%)	68	1 (1.5%)	1.000
Chloroquine	425	4 (0.9%)	357	1 (0.3%)	68	3 (4.4%)	0.014
Antiarrhythmics	425	3 (0.7%)	357	2 (0.6%)	68	1 (1.5%)	0.408
Barbiturates	425	1 (0.2%)	357	1 (0.3%)	68	0 (0%)	1.000
Non-pharmaceutical (toxics)	425	103 (24.2%)	357	80 (22.4%)	68	23 (33.8%)	0.044
Caustic	425	43 (10.1%)	357	37 (10.4%)	68	6 (8.8%)	0.699
Herbicide	425	27 (6.4%)	357	12 (3.4%)	68	15 (22.1%)	0.000
Paraquat	425	22 (5.2%)	357	7 (2%)	68	15 (22.1%)	0.000
Cocaine	425	8 (1.9%)	357	8 (2.2%)	68	0 (0%)	0.365
Oxalic acid	424	6 (1.4%)	356	6 (1.7%)	68	0 (0%)	0.596
Ammonia	425	6 (1.4%)	357	5 (1.4%)	68	1 (1.5%)	1.000
Rodenticide	280	4 (1.4%)	231	3 (1.3%)	49	1 (2%)	0.539
Insecticide	425	4 (0.9%)	357	4 (1.1%)	68	0 (0%)	1.000
Organophosphorus	425	4 (0.9%)	357	3 (0.8%)	68	1 (1.5%)	0.503
Petroleum product	425	4 (0.9%)	357	4 (1.1%)	68	0 (0%)	1.000
Cannabis	425	4 (0.9%)	357	4 (1.1%)	68	0 (0%)	1.000
Ciguatoxin	425	2 (0.5%)	357	2 (0.6%)	68	0 (0%)	1.000
Aldicarbe	425	1 (0.2%)	357	1 (0.3%)	68	0 (0%)	1.000
Ethylene-glycol	425	1 (0.2%)	357	1 (0.3%)	68	0 (0%)	1.000
Crack	425	1 (0.2%)	357	1 (0.3%)	68	0 (0%)	1.000
Plants	425	1 (0.2%)	357	1 (0.3%)	68	0 (0%)	1.000

Values indicate the number of patients for whom data were recorded (Nb), the median and interquartile range, or numbers and percentages.

**Table 3:** Clinical presentation at admission to the emergency department.

	Total		PSS 1 or 2		PSS 3 or 4		*p*
	Nb	Result	Nb	Result	Nb	Result	
Temperature (°C)	382	36.8 (36.5–37.1)	323	36.8 (36.5–37.1)	59	36.7 (36.4–37.2)	0.799
Hypothermia	382	8 (2.1%)	323	3 (0.9%)	59	5 (8.5%)	0.003
Fever	382	5 (1.3%)	323	3 (0.9%)	59	2 (3.4%)	0.172
Respiratory rate (cycles/min)	312	16 (12–22)	259	16 (12–21)	53	20 (12–24)	0.028
Bradypnea	307	92 (30%)	257	78 (30.4%)	50	14 (28%)	0.740
Heart rate (beats/min)	422	86 (74–99)	355	86 (74–99)	67	85 (74–103)	0.849
Bradycardia	422	2 (0.5%)	355	2 (0.6%)	67	0 (0%)	1.000
Tachycardia	422	104 (24.6%)	355	84 (23.7%)	67	20 (29.9%)	0.281
Circulatory shock	423	16 (3.8%)	355	0 (0%)	68	16 (26.5%)	0.000
Glasgow coma scale	423	15 (12–15)	355	15 (13–15)	68	13 (7–15)	0.000
Coma	423	41 (9.7%)	355	10 (2.8%)	68	31 (45.6%)	0.000
Seizures	423	6 (1.4%)	355	1 (0.3%)	68	5 (7.4%)	0.000
Digestive disorders	424	211 (49.8%)	356	181 (50.8%)	68	30 (44.1%)	0.309
Conjunctivitis	423	5 (1.2%)	355	5 (1.4%)	68	0 (0%)	1.000

Values indicate the number of patients for whom data were recorded (Nb), the median and interquartile range, or numbers and percentages.

**Table 4:** Biological data recorded at admission to the emergency department.

	Total		PSS 1 or 2		PSS 3 or 4		*p*
	Nb	Result	Nb	Result	Nb	Result	
pH	85	7.38 (7.35–7.42)	57	7.4 (7.37–7.42)	28	7.36 (7.31–7.41)	0.012
Alkaline reserve (mmol/L)	389	24 (22–26)	321	24 (22.6–26)	68	22.6 (18.1–25.3)	0.000
Metabolic acidosis	388	113 (29.1%)	321	80 (24.9%)	67	33 (49.3%)	0.000
Lactates (mmol/L)	93	2 (1–3.2)	70	1.9 (1–2.9)	23	3.7 (1.4–7)	0.022
Hyperlactatemia	93	0 (0–1)	70	31 (44.3%)	23	13 (56.5%)	0.308
Venous glycemia (mmol/L)	368	5.2 (4.6–6.1)	309	5.2 (4.6–6)	59	5.6 (4.6–8.3)	0.023
Hypoglycemia	368	18 (4.9%)	309	15 (4.9%)	59	3 (5.1%)	1.000
Hyperglycemia	368	41 (11.1%)	309	24 (7.8%)	59	17 (28.8%)	0.000
Natremia (mmol/L)	421	138 (136–141)	353	138 (136–140)	68	139 (137–141)	0.543
Hyponatremia	421	52 (12.4%)	353	43 (12.2%)	68	9 (13.2%)	0.809
Kalemia (mmol/L)	404	3.7 (3.4–4.0)	339	3.7 (3.4–4.1)	65	3.8 (3.4–4)	0.862
Hypokalemia	404	107 (26.5%)	339	88 (26%)	65	19 (29.2%)	0.584
Hyperkalemia	404	7 (1.7%)	339	0 (0%)	65	7 (10.8%)	0.000
Calcemia (mmol/L)	416	2.3 (2.2–2.5)	348	2.3 (2.2–2.5)	68	2.29 (2.2–2.4)	0.013
Magnesemia (mmol/L)	11	0.7 (0.7–0.9)	5	0.7 (0.7–0.9)	6	0.7 (0.7–1)	0.848
Phosphoremia (mmol/L)	13	0.9 (0.7–1.2)	5	1.1 (0.9–1.2)	8	0.9 (0.7–1.4)	0.825
Creatinine (µmol/L)	420	69 (58–85)	352	68 (57–81)	68	81 (66–125)	0.000
Acute renal failure	420	42 (10%)	352	23 (6.5%)	68	18 (26.5%)	0.000
Aspartate aminotransferase (IU/L)	381	16 (12–25)	320	16 (11–24)	61	21 (12–42)	0.009
Alanine aminotransferase (IU/L)	370	21 (16–28)	310	21 (16–27)	60	25 (18–51)	0.013
Hepatic cytolysis	381	45 (11.8%)	320	29 (9.1%)	61	16 (26.2%)	0.000
Creatinine phosphokinase (IU/L)	266	92 (65–192)	221	92 (65–186)	45	92 (66–262)	0.470
Rhabdomyolysis	264	19 (7.2%)	220	11 (5%)	44	8 (18.2%)	0.002
Troponin (µg/L)	40	0.01 (0.005–0.028)	28	0.006 (0.005–0.011)	12	0.017 (0.011–0.06)	0.016
Platelets (G/L)	414	264 (219–321)	347	270 (220–324)	67	249 (217–283)	0.027
Thrombocytopenia	413	15 (3.6%)	347	9 (2.6%)	66	6 (9.1%)	0.020
Prothrombin rate (%)	322	87 (77–96)	267	88 (79–97)	55	80 (69–89)	0.000

Values indicate the number of patients for whom data were recorded (Nb), the median and interquartile range, or numbers and percentages.

**Table 5:** Therapeutic management at admission to the emergency department and during hospital stay.

	Total		PSS 1 or 2		PSS 3 or 4		*p*
	**Nb**	**Result**	**Nb**	**Result**	**Nb**	**Result**	
Activated charcoal	425	119 (28%)	357	102 (28.6%)	68	17 (25%)	0.548
Gastric lavage	425	50 (11.8%)	357	32 (9%)	68	18 (26.5%)	0.000
Antidotes	425	191 (44.9%)	357	165 (46.2%)	68	26 (38.2%)	0.225
Mechanical ventilation (MV)	425	15 (3.5%)	357	0 (0%)	68	15 (22.1%)	0.000
Duration of MV (days)	15	4 (1–9)	-	-	15	4 (1–9)	-
Vasopressors	425	18 (4.2%)	357	0 (0%)	68	18 (26.5%)	0.000
Dialysis	425	3 (0.7%)	357	0 (0%)	68	3 (4.4%)	0.004

Values indicate the number of patients for whom data were recorded (Nb), the median and interquartile range (IQR), or numbers and percentages.

**Table 6:** Factors associated with severe poisoning in multivariate analysis.

	*p*	OR	95% CI	
			Lower	Upper
Paracetamol	0.036	0.324	0.113	0.931
Choroquine	0.017	18.274	1.665	200.561
Neuroleptics	0.009	3.893	1.409	10.753
Paraquat	0.012	5.472	1.452	20.618
Metabolic acidosis	0.048	2.109	1.005	4.424
Hyperglycemia (>5.5 mmol/L)	0.049	2.404	1.002	5.766

## Data Availability

All data supporting reported results are available from the corresponding author upon request.

## References

[1] Zhang Y., Yu B., Wang N., Li T. (2018). Acute Poisoning in Shenyang, China: A Retrospective and Descriptive Study from 2012 to 2016. BMJ Open.

[2] Resiere D., Kallel H., Oxybel O., Chabartier C., Florentin J., Brouste Y., Gueye P., Megarbane B., Mehdaoui H. (2020). Clinical and Epidemiological Characteristics of Severe Acute Adult Poisoning Cases in Martinique: Implicated Toxic Exposures and Their Outcomes. Toxics.

[3] Getie A., Belayneh Y.M. (2020). A Retrospective Study of Acute Poisoning Cases and Their Management at Emergency Department of Dessie Referral Hospital, Northeast Ethiopia. Drug Healthc. Patient Saf..

[4] Singh S.P., Aggarwal A.D., Oberoi S.S., Aggarwal K.K., Thind A.S., Bhullar D.S., Walia D.S., Chahal P.S. (2013). Study of Poisoning Trends in North India—A Perspective in Relation to World Statistics. J. Forensic Leg. Med..

[5] Baud F., Garnier R. (2017). Toxicologie Clinique.

[6] WHO Poison Control and Unintentional Poisoning. https://www.who.int/data/gho/data/themes/topics/indicator-groups/poison-control-and-unintentional-poisoning.

[7] Islambulchilar M., Islambulchilar Z., Kargar-Maher M.H. (2009). Acute Adult Poisoning Cases Admitted to a University Hospital in Tabriz, Iran. Hum. Exp. Toxicol..

[8] Albano G.D., Malta G., La Spina C., Rifiorito A., Provenzano V., Triolo V., Vaiano F., Bertol E., Zerbo S., Argo A. (2022). Toxicological Findings of Self-Poisoning Suicidal Deaths: A Systematic Review by Countries. Toxics.

[9] Jesslin J., Adepu R., Churi S. (2010). Assessment of Prevalence and Mortality Incidences Due to Poisoning in a South Indian Tertiary Care Teaching Hospital. Indian J. Pharm. Sci..

[10] Teklemariam E., Tesema S., Jemal A. (2016). Pattern of Acute Poisoning in Jimma University Specialized Hospital, South West Ethiopia. World J. Emerg. Med..

[11] Baydin A., Yardan T., Aygun D., Doganay Z., Nargis C., Incealtin O. (2005). Retrospective Evaluation of Emergency Service Patients with Poisoning: A 3-Year Study. Adv. Ther..

[12] Villa A., Cochet A., Guyodo G. (2008). Poison episodes reported to French poison control centers in 2006. Rev. Prat..

[13] Boedeker W., Watts M., Clausing P., Marquez E. (2020). The Global Distribution of Acute Unintentional Pesticide Poisoning: Estimations Based on a Systematic Review. BMC Public Health.

[14] Al-Daghastani T., Naser A.Y. (2022). Hospital Admission Profile Related to Poisoning by, Adverse Effect of and Underdosing of Psychotropic Drugs in England and Wales: An Ecological Study. Saudi Pharm. J..

[15] Vuolo M., Frizzell L.C., Kelly B.C. (2021). Trends in Psychotropic-Drug-Implicated Mortality: Psychotropic Drugs as a Contributing but Non-Underlying Cause of Death. Drug Alcohol Depend..

[16] Coben J.H., Davis S.M., Furbee P.M., Sikora R.D., Tillotson R.D., Bossarte R.M. (2010). Hospitalizations for Poisoning by Prescription Opioids, Sedatives, and Tranquilizers. Am. J. Prev. Med..

[17] Armstrong T.M., Davies M.S., Kitching G., Waring W.S. (2012). Comparative Drug Dose and Drug Combinations in Patients That Present to Hospital Due to Self-Poisoning. Basic Clin. Pharmacol. Toxicol..

[18] Rani F., Murray M.L., Byrne P.J., Wong I.C.K. (2008). Epidemiologic Features of Antipsychotic Prescribing to Children and Adolescents in Primary Care in the United Kingdom. Pediatrics.

[19] Hirschtritt M.E., Slama N., Sterling S.A., Olfson M., Iturralde E. (2021). Psychotropic Medication Prescribing during the COVID-19 Pandemic. Medicine.

[20] Paulose-Ram R., Jonas B.S., Orwig D., Safran M.A. (2004). Prescription Psychotropic Medication Use among the U.S. Adult Population: Results from the Third National Health and Nutrition Examination Survey, 1988–1994. J. Clin. Epidemiol..

[21] Vargas A., Ormseth G., Seifi A. (2020). Gender and Psychotropic Poisoning in the USA. J. Neurol. Res..

[22] CIM-10 Version: 2008. https://icd.who.int/browse10/2008/fr.

[23] Cairns R., Buckley N.A. (2017). The Poisoning Severity Score: If It Did Not Exist, We Would Have to Invent It. J. Med. Toxicol..

[24] Khwaja A. (2012). KDIGO Clinical Practice Guidelines for Acute Kidney Injury. Nephron Clin. Pract..

[25] Gummin D.D., Mowry J.B., Spyker D.A., Brooks D.E., Beuhler M.C., Rivers L.J., Hashem H.A., Ryan M.L. (2019). 2018 Annual Report of the American Association of Poison Control Centers’ National Poison Data System (NPDS): 36th Annual Report. Clin. Toxicol..

[26] Tooplate NPIS. https://www.npis.org/index.html.

[27] Sorge M., Weidhase L., Bernhard M., Gries A., Petros S. (2015). Self-Poisoning in the Acute Care Medicine 2005–2012. Anaesthesist.

[28] Lindqvist E., Edman G., Hollenberg J., Nordberg P., Ösby U., Forsberg S. (2017). Intensive Care Admissions Due to Poisoning. Acta Anaesthesiol. Scand..

[29] Maignan M., Pommier P., Clot S., Saviuc P., Debaty G., Briot R., Carpentier F., Danel V. (2014). Deliberate Drug Poisoning with Slight Symptoms on Admission: Are There Predictive Factors for Intensive Care Unit Referral? A Three-Year Retrospective Study. Basic Clin. Pharmacol. Toxicol..

[30] Henderson A., Wright M., Pond S.M. (1993). Experience with 732 Acute Overdose Patients Admitted to an Intensive Care Unit over Six Years. Med. J. Aust..

[31] Kapur N., House A., Creed F., Feldman E., Friedman T., Guthrie E. (1998). Management of Deliberate Self Poisoning in Adults in Four Teaching Hospitals: Descriptive Study. BMJ.

[32] Liisanantti J.H., Ohtonen P., Kiviniemi O., Laurila J.J., Ala-Kokko T.I. (2011). Risk Factors for Prolonged Intensive Care Unit Stay and Hospital Mortality in Acute Drug-Poisoned Patients: An Evaluation of the Physiologic and Laboratory Parameters on Admission. J. Crit. Care.

[33] Sharma R., Neelanjana, Rawat N., Panwar N. (2019). Mortality and Morbidity Associated with Acute Poisoning Cases in North-East India: A Retrospective Study. J. Fam. Med. Prim. Care.

[34] Prashar A., Ramesh M. (2018). Assessment of Pattern and Outcomes of Pesticides Poisoning in a Tertiary Care Hospital. Trop. Med. Int. Health.

[35] Liu S., Ling L., Ma J., Yuan H., Guo Z., Feng Q., Xia X. (2023). Trends and Profiles of Acute Poisoning Cases: A Retrospective Analysis. Front. Public Health.

[36] Ahmadi A., Pakravan N., Ghazizadeh Z. (2010). Pattern of Acute Food, Drug, and Chemical Poisoning in Sari City, Northern Iran. Hum. Exp. Toxicol..

[37] Apata J., Pennap D., Ma Y., Mosholder A. (2023). Suicide Poisoning Mortality: A Comparison of the National Poison Data System and Centers for Disease Control National Dataset. Inj. Prev..

[38] Al-Jelaify M., AlHomidah S. (2021). The Individualized Management Approach for Acute Poisoning. Adv. Pharmacol. Pharm. Sci..

[39] Veale D.J.H., Wium C.A., Müller G.J. (2013). Toxicovigilance. II: A Survey of the Spectrum of Acute Poisoning and Current Practices in the Initial Management of Poisoning Cases Admitted to South African Hospitals. S. Afr. Med. J..

[40] Burillo-Putze G., Munne P., Dueñas A., Pinillos M.A., Naveiro J.M., Cobo J., Alonso J., Clinical Toxicology Working Group, Spanish Society of Emergency Medicine (SEMESTOX) (2003). National Multicentre Study of Acute Intoxication in Emergency Departments of Spain. Eur. J. Emerg. Med..

[41] Brandenburg R., Brinkman S., de Keizer N.F., Meulenbelt J., de Lange D.W. (2014). In-Hospital Mortality and Long-Term Survival of Patients with Acute Intoxication Admitted to the ICU*. Crit. Care Med..

[42] [Multisource observatory of acute intoxications in Île-de-France: An exploratory study]. https://www.santepubliquefrance.fr/notices/observatoire-multisources-des-intoxications-aigues-en-ile-de-france-une-etude-exploratoire.

[43] WHO Suicide (2023). https://www.who.int/news-room/fact-sheets/detail/suicide.

[44] Graafsma T., Kerkhof A., Gibson D., Badloe R., van de Beek L.M. (2006). High Rates of Suicide and Attempted Suicide Using Pesticides in Nickerie, Suriname, South America. Crisis.

[45] Elenga N., Merlin C., Le Guern R., Kom-Tchameni R., Ducrot Y.-M., Pradier M., Ntab B., Dinh-Van K.-A., Sobesky M., Mathieu D. (2018). Clinical Features and Prognosis of Paraquat Poisoning in French Guiana: A Review of 62 Cases. Medicine.

[46] Donaher S.E., Van den Hurk P. (2023). Ecotoxicology of the Herbicide Paraquat: Effects on Wildlife and Knowledge Gaps. Ecotoxicology.

[47] Jafari-Nozad A.M., Jafari A., Aschner M., Farkhondeh T., Samarghandian S. (2023). Curcumin Combats against Organophosphate Pesticides Toxicity: A Review of the Current Evidence and Molecular Pathways. Curr. Med. Chem..

[48] [Senate bans use of the herbicide Paraquat in France]. https://www.senat.fr/questions/base/2004/qSEQ040712949.html.

[49] Flechel A., Jolivet A., Boukhari R., Misslin-Tritsch C., Manca M.F., Wiel E., Megarbane B., Pousset F. (2018). Paraquat Poisoning in Western French Guyana: A Public Health Problem Persisting Ten Years after Its Withdrawal from the French Market. Eur. Rev. Med. Pharmacol. Sci..

[50] Kervégant M., Schmitt C., Martin E., Merigot L., Tichadou L., Bonnet P., De Haro L. (2013). Intoxications Au Paraquat En Guyane Française: Utilisation Persistante Lors de Comportements Suicidaires En Outre-Mer. Ann. Toxicol. Anal..

[51] Boucaud-Maitre D., Rambourg M.-O., Sinno-Tellier S., Puskarczyk E., Pineau X., Kammerer M., Bloch J., Langrand J. (2019). Human Exposure to Banned Pesticides Reported to the French Poison Control Centers: 2012–2016. Environ. Toxicol. Pharmacol..

[52] Andrade C., Villers A., Balent G., Bar-Hen A., Chadoeuf J., Cylly D., Cluzeau D., Fried G., Guillocheau S., Pillon O. (2021). A Real-World Implementation of a Nationwide, Long-Term Monitoring Program to Assess the Impact of Agrochemicals and Agricultural Practices on Biodiversity. Ecol. Evol..

[53] Bismuth C., Garnier R., Baud F.J., Muszynski J., Keyes C. (1990). Paraquat Poisoning: An Overview of the Current Status. Drug Saf..

[54] Wilks M.F., Fernando R., Ariyananda P.L., Eddleston M., Berry D.J., Tomenson J.A., Buckley N.A., Jayamanne S., Gunnell D., Dawson A. (2008). Improvement in Survival after Paraquat Ingestion Following Introduction of a New Formulation in Sri Lanka. PLoS Med..

[55] Eddleston M. (2022). Evidence for the Efficacy of the Emetic PP796 in Paraquat SL20 Formulations–a Narrative Review of Published and Unpublished Evidence. Clin. Toxicol..

[56] Myung W., Lee G.-H., Won H.-H., Fava M., Mischoulon D., Nyer M., Kim D.K., Heo J.-Y., Jeon H.J. (2015). Paraquat Prohibition and Change in the Suicide Rate and Methods in South Korea. PLoS ONE.

[57] Camargo E.R., Zapiola M.L., de Avila L.A., Garcia M.A., Plaza G., Gazziero D., Hoyos V. (2020). Current Situation Regarding Herbicide Regulation and Public Perception in South America. Weed Sci..

[58] Pathak V.M., Verma V.K., Rawat B.S., Kaur B., Babu N., Sharma A., Dewali S., Yadav M., Kumari R., Singh S. (2022). Current Status of Pesticide Effects on Environment, Human Health and It’s Eco-Friendly Management as Bioremediation: A Comprehensive Review. Front. Microbiol..

[59] Hoffmann V., Paul B., Falade T., Moodley A., Ramankutty N., Olawoye J., Djouaka R., Lekei E., de Haan N., Ballantyne P. (2022). A One Health Approach to Plant Health. CABI Agric. Biosci..

[60] Fernández-Ibáñez A., Ugalde-Herrá R., Rodríguez-Getino J.Á., García Casas J.B., Díaz-Suárez J.C. (2021). Epidemiology of Acute Poisoning by Substances of Abuse in the Emergency Department. Descriptive Study in District IV of Asturias. Adicciones.

[61] Léon C., Chan-Chee C., du Roscoät E. (2019). Santé Publique France Health Barometer 2017: Suicidal Attempts and Suicidal Ideation among the 18–75 Years-Old. Bull. Épidémiologique Hebd..

[62] Suicide Acts in 8 States: Incidence and Case Fatality Rates by Demographics and Method–PMC. https://www.ncbi.nlm.nih.gov/pmc/articles/PMC1446422/.

[63] Bertolote J.M., Fleischmann A., De Leo D., Bolhari J., Botega N., De Silva D., Tran Thi Thanh H., Phillips M., Schlebusch L., Värnik A. (2005). Suicide Attempts, Plans, and Ideation in Culturally Diverse Sites: The WHO SUPRE-MISS Community Survey. Psychol. Med..

[64] Guarmit B., Brousse P., Lucarelli A., Donutil G., Cropet C., Mosnier E., Travers P., Nacher M. (2018). Descriptive Epidemiology of Suicide Attempts and Suicide in the Remote Villages of French Guiana. Soc. Psychiatry Psychiatr. Epidemiol..

[65] Collados-Ros A., Torres-Sánchez C., Pérez-Cárceles M.D., Luna A., Legaz I. (2022). Suicidal Behavior and Its Relationship with Postmortem Forensic Toxicological Findings. Toxics.

[66] Pacot R., Garmit B., Pradem M., Nacher M., Brousse P. (2018). The Problem of Suicide among Amerindians in Camopi-Trois Sauts, French Guiana 2008–2015. BMC Psychiatry.

[67] Sinha S.N., Kumpati R.K., Ramavath P.N., Sangaraju R., Gouda B., Chougule P. (2022). Investigation of Acute Organophosphate Poisoning in Humans Based on Sociodemographic and Role of Neurotransmitters with Survival Study in South India. Sci. Rep..

[68] Aguilón-Leiva J.J., Tejada-Garrido C.I., Echániz-Serrano E., Mir-Ramos E., Torres-Pérez A.M., Lafuente-Jiménez A., Martínez-Soriano M., Santolalla-Arnedo I., Czapla M., Smereka J. (2022). Clinical and Sociodemographic Profile of Acute Intoxications in an Emergency Department: A Retrospective Cross-Sectional Study. Front. Public Health.

[69] Alhaboob A.A. (2021). Sociodemographic Characteristics and Risk Factors for Childhood Poisoning Reported by Parents at a Tertiary Care Teaching Hospital. Cureus.

